# Use of Chinese Medicine and Subsequent Surgery in Women with Uterine Fibroid: A Retrospective Cohort Study

**DOI:** 10.1155/2012/617918

**Published:** 2012-10-16

**Authors:** Shan-Yu Su, Chih-Hsin Muo, Donald E. Morisky

**Affiliations:** ^1^Department of Chinese Medicine, China Medical University Hospital, No. 2 Yuh-Der Road, Taichung 40447, Taiwan; ^2^School of Post-Baccalaureate Chinese Medicine, College of Chinese Medicine, China Medical University, Taichung 40402, Taiwan; ^3^Department of Public Health, China Medical University, Taichung 40402, Taiwan; ^4^Management Office for Health Data, China Medical University Hospital, Taichung 40402, Taiwan; ^5^Department of Community Health Sciences, UCLA Fielding School of Public Health, Los Angeles, CA 90095-1772, USA

## Abstract

*Background*. Chinese medicine (CM) has been used to relieve symptoms relevant to uterine fibroids. *Objective*. This study investigated the association between the use of CM and the incidence of uterine surgery in women with uterine fibroids. *Subjects and Methods*. This retrospective cohort study extracted records for 16,690 subjects diagnosed with a uterine fibroid between 2000 and 2003 from the National Health Insurance reimbursement database. The risk factors for surgery were examined via Cox proportional hazard analysis, and the difference in incidence of surgery between CM users and nonusers was compared using incidence rate ratios (IRRs) derived from Poisson's models. *Results*. After an average follow-up period of 4.5 years, the cumulative incidence of uterine surgery was significantly lower in CM users than CM nonusers (*P* < 0.0001). Compared to CM nonusers, CM users were more unlikely to undergo uterine surgery (adjusted hazard ratio = 0.18, 95% confidence interval (CI) = 0.17, 0.19). The incidence of surgery in CM users was dramatically different from that for CM nonusers (IRR = 0.17, 95% CI = 0.16, 0.18). *Conclusion*. The risk of uterine surgery among fibroid patients who used CM was significantly decreased, implying an effective treatment of fibroid-related symptoms provided by CM.

## 1. Introduction

Uterine fibroids, also known as leiomyoma, are the most commonly occurring benign tumors of the female reproductive system, with a cumulative incidence of more than 60% in women over the age of 45 years [1]. They are the leading indication for hysterectomy all over the world and are associated with a substantial economic impact on health care systems, including associated costs of $4–9 billion per year in the United States alone [2]. These tumors grow frequently in women of reproductive age, regressing after menopause [3], and can cause various symptoms including infertility, pregnancy complications [4], pelvic pain [5], and abnormal or heavy bleeding that can lead to anemia [6].

Surgery has traditionally been the gold standard for the treatment of symptomatic uterine fibroids. Hysterectomy is indicated in women who have completed childbearing, particularly in those who are expected to go into menopause soon, while myomectomy is indicated in women who wish to preserve their fertility [7]. For asymptomatic patients, on the other hand, serial followups (without surgery) to monitor the size of the tumor and check for related symptoms are advisable [8]. Surgery is an invasive treatment that can cause even more severe complications than a fibroid itself. Potential short-term complications of surgery include febrile morbidity, blood loss, and organ injuries [9], while possible long-term complications, such as fistula formation, adhesion, and sexual dysfunctions [10], can last for many years, even beyond menopause, when a uterine leiomyoma is no longer a threat to health [3]. Consequently, alternative treatments to surgery for the management of fibroid-related symptoms have been sought and evaluated for years to minimize surgery in patients [11].

In Asian countries, Chinese medicine (CM) is one of the most commonly used complementary alternative medicines and has been reported to be used more by females than by males around the world [12–16]. In Taiwan, CM has been covered by the National Health Insurance (NHI) system since 1995 [17, 18]. According to the NHI database, diseases of the female reproductive system, including menstruation disorders, abnormal bleeding, and noninflammatory disorders of female genital organs, are on the list of the top twenty most common diseases for which patients utilize CM [19]. The high utilization rate of CM for these symptoms, all of which are potentially related to uterine fibroids, implies the possibility that CM might treat those symptoms and, in turn, reduce surgery among patients with uterine fibroids. 

This population-based retrospective cohort study used a national insurance reimbursement database to investigate the association between the use of CM and the incidence of uterine surgery in women with uterine fibroids. The results provide population-based evidence for the benefit of CM in women with uterine fibroids.

## 2. Materials and Methods

### 2.1. Study Subjects

This study used the Longitudinal Health Insurance Database 2000 (LHID2000), which is a part of the National Health Insurance Research Database set up by Taiwan's National Health Research Institutes. This database contains chronological information on one million randomly selected individuals who were beneficiaries from 1996 to 2000. There are no differences in gender and age between the beneficiaries in the LHID2000 and the beneficiaries in the entire NHI database. The beneficiary information includes gender, birth date, income, occupation status, area of registration, all medical claims for inpatient and outpatient care, the dates of visits, and up to three diagnostic codes in the International Classification of Diseases, 9th Revision, Clinical Modification (ICD-9-CM) of these beneficiaries from 1996 to 2009.

A total of 16,848 women were diagnosed with uterine fibroid (ICD-9-CM 218) from 2000 to 2003 in outpatient visits. The diagnosis date was used as the entry date and surgery was defined as the outcome. There were 158 women who were excluded because they had undergone uterine surgery before the entry date. The remaining 16,690 women were selected as research subjects and divided into CM users and CM nonusers according to their use (or nonuse) of orally administered CM between the entry date and endpoint date. The endpoint date was defined as the date of surgery, death, withdrawal from the insurance program, or December 31, 2009.

The examined variables were sociodemographic factors, including age (<20, 20–29, 30–39, 40–49, and ≥50 years), income level (<564.3, 564.3–656.5, 656.6–779.6, and ≥779.7 US$ per month based on quartile), occupation status (white collar, blue collar, and others), and registered location (northern, central, southern, eastern, and island), and fibroid-related comorbidities, including excessive menstruation (ICD-9-CM 626.2), iron-deficiency anemia (ICD-9-CM 280), dysmenorrhea (ICD-9-CM 625.3), and infertility (ICD-9-CM 628 and 628.3).

### 2.2. Statistical Analysis

The chi-square test and *t*-test were used to assess differences for categorical and continuous variables between CM users and CM nonusers, respectively. Cox proportional regression was used to estimate the hazard ratio (HR) and its 95% confidence intervals (CIs) for undergoing surgery. The Kaplan-Meier method was used to plot the cumulative incidence, and the log-rank test was used to test the difference in cumulative rates between CM users and nonusers. Adjusted models were controlled for age, occupation, area, excessive menstruation, iron-deficiency anemia, and dysmenorrhea. The incidence rate (IR) for surgery (per 1000 person-years) was also calculated. The association between the use of CM and uterine surgery was estimated by using the incidence rate ratio (IRR) and the corresponding 95% CI by Poissons distribution model. All statistical analyses were performed using SAS software, version 9.1 (SAS Institute Inc., Carey, NC), and the significance level was set at a two-tailed *P* value of less than 0.05.

## 3. Results

### 3.1. Characteristics of CM Users and CM Nonusers in Fibroid Patients

Among the 16,690 females with a uterine fibroid diagnosis from 2000 to 2003, 12,238 (73.3%) used CM between the diagnosis and endpoint date. The mean age of CM users (41.6 years) was lower than that of CM nonusers (42.9 years). Significantly, the proportion of patients among CM users who were young (under 40), white collar, and registered their insurance in Central Taiwan was higher than that among CM nonusers. In terms of comorbidity, CM users were more likely to have fibroid-related comorbidities, including excessive menstruation, anemia, and dysmenorrhea (Table 1).

### 3.2. Risk Factors for Uterine Surgery among Fibroid Patients

At the end of the observation, 22% of the patients (*n* = 3681) received surgery of the uterus, including myomectomy and hysterectomy. Compared to nonusers, CM users were significantly more unlikely to undergo surgery after adjustment for socio-demographic factors (age, occupation, and area) and comorbid covariates (excessive menstruation, iron-deficiency anemia, and dysmenorrhea), with an HR of 0.18 (Table 2). The adjusted HRs for surgery were more than double among patients between 30 and 50 years of age compared to those who were under 30 years old. Adjusted HRs were also higher among patients who were blue collar, registered in Central and Southern Taiwan, and whose diseases were comorbid with excessive menstruation, iron-deficiency anemia, and dysmenorrhea. The mean observation time in this cohort was 4.5 years.

### 3.3. Incidence of Surgery among CM Users and CM Nonusers

**Figure 1:**
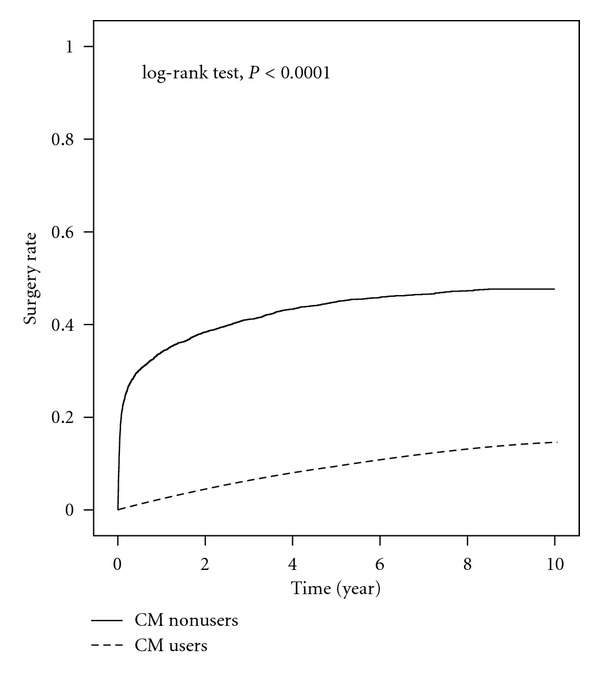
Kaplan-Meier analysis for cumulative incidence of uterine surgery between CM users and CM nonusers.

At the end of observation, 8.7% of CM users and 46.6% of CM nonusers have undergone surgery. Kaplan-Meier's analysis showed a significant difference in cumulative incidence of surgery between CM users and nonusers (*P* < 0.0001, Figure 1). This difference developed rapidly in the first several months after the diagnosis of a uterine fibroid.

The overall IR for surgery among CM users was 17.7 per 1,000 fibroid patients, while that among CM nonusers was 103.7 per 1,000. Poisson's regression modeling revealed that the IRs for surgery were lower among CM users in all demographic subgroups except for those younger than 20 years of age. In terms of fibroid-related comorbidity, the IRs were also lower for CM users than for CM nonusers whether patients had excessive menstruation, anemia, and dysmenorrhea or not, while the IRs were similar between CM users and nonusers among patients with infertility (Table 3).

In light of the rapid development of the difference in the cumulative incidence of surgery between the two groups, time lag stratification was performed to rule out the possibility that rapid decisions in favor of surgery biased the results (Table 4). Adjusted HRs for surgery remained lower in CM users than in CM nonusers even after the deletion of subjects with a diagnosis-to-surgery period of less than three months to five years, but the adjusted HR for surgery in CM users increased from 0.33 to 0.79 compared to nonusers. 

## 4. Discussion

This retrospective cohort study investigated the relationship between the use of CM and the incidence of uterine surgery in women with uterine fibroids using an NHI database documenting medical claims from 1996 to 2009. After an average follow-up time of 4.5 years, the data shows that patients who received CM had a significantly lower risk of uterine surgery compared to patients who did not receive CM treatment. Moreover, the incidence of surgery in CM nonusers was more than five times higher than in CM users. The low surgery incidence in CM users was not affected by age, income, occupational status, area of insurance registration, or comorbidities including excessive menstruation, iron-deficiency anemia, and dysmenorrhea.

The strength of the present study is that the database we used was from the NHI, which is a government-run, single-payer national health insurance program that insures over 97% of citizens and over 99% of health-care institutes [20, 21]; this rendered the present study representative of the general population, thereby offering a comprehensive picture of the risks of surgery in fibroid patients. The data revealed a lower surgery incidence in fibroid patients who used CM compared to those who did not use CM, implying that Taiwanese women with uterine fibroids benefited sufficiently from CM to avoid surgery. This study further suggested that CM might provide an effective alternative therapy to surgery for uterine fibroids.

Several studies have reported sociodemographic trends regarding the use of CM in women. CM users have been reported to be younger than CM nonusers when it comes to women with constipation [22], insomnia [23], and breast cancer [24], although the primary age group varies from disease to disease. The present study supported such findings by showing that patients under 40 with uterine fibroids were more likely to use CM. In terms of income, education, and occupational status, studies that have investigated female-specific diseases, including breast cancer and gynecological malignancies, have reported that users of CM and complementary medicines tend to be highly educated, have high incomes, and are more likely to be employed by the government, schools, enterprises, and institutions [24, 25]. The data of the present study, meanwhile, showed that while CM users tended to be white collar instead of blue collar workers, there were no differences in income between CM users and nonusers. The present study also found that there was a higher proportion of patients who registered their insurance in Central Taiwan among CM users than among CM nonusers, a finding being attributed to the high density of Chinese medical institutes per person in Central Taiwan [12, 24]. When taking into account comorbidities, it appeared that fibroid patients with relevant comorbidities tended to use CM. Since suffering from disease is one of the factors that has been reported to positively and directly influence the purchasing behavior of CM outpatients [26], the existence of symptoms related to uterine fibroids might be one of the factors that drove patients to utilize CM.

Analysis of the risk factors for surgery revealed that patients who are more than 30 years of age, blue collar, and registered in the Central and Southern regions were more likely to undergo surgery. Moreover, fibroid-related symptoms, including excessive menstruation, anemia, and dysmenorrhea, were also risk factors for surgery (although infertility was not related to surgery). These risk factors had HRs ranging from 1.24 to 2.54. On the other hand, the adjusted HR for surgery in CM users was 0.18 compared to nonusers, indicating that CM nonusers were 5.5 times more at risk of surgery than CM users. Moreover, the overall IR of surgery for CM users was 0.17 times that of CM nonusers. Both results imply that the use of CM was a protective factor against surgery in fibroid patients. In the stratified analysis, the IRRs of surgery in CM users for all the subgroups of sociodemographic status and for almost all the subgroups of comorbidities were lower than 0.25, implying that the protective effect of CM was strong enough to protect almost all the subgroups for sociodemographic and comorbid status. Only in patients with infertility did the difference in IR between CM users and nonusers not exist. The reason was speculated to be that hysterectomy is contraindicated for women who want to preserve their fertility, and therefore patients with infertility did not tend to receive surgery regardless of whether they utilized CM or not.

Although there was a big difference in the cumulative incidence of surgery between CM users and nonusers, Kaplan-Meier's analysis indicated that the difference increased rapidly in the first few months after diagnosis. Results for a time lag stratification revealed that even after the deletion of subjects with a diagnosis-to-surgery period of less than three months to five years, the differences in IR between the two groups still existed, indicating that there were still protective effects from CM in patients who had been diagnosed for five years. However, the adjusted HR for surgery increased from 0.33 to 0.79 with time lag periods from three months to five years, indicating that the protective effects were weakened in patients who had a long diagnosis-to-surgery duration. We speculate that the increased proportion of asymptomatic patients in groups of long diagnosis-to-surgery duration caused the weakening of CM protection. Surgery is not indicated in asymptomatic patients; therefore, those who were asymptomatic would not undergo surgery whether they took CM treatment or not.

Clinical trials regarding the effects of CM on the outcome of uterine fibroids are very limited. A small randomized controlled trial with 25 women in total, using strict randomization methods and data management, showed that fibroids shrank after treatment with a medicinal formulation of Nona Roguy for six months; the shrinkage of fibroids for the CM group was comparable to that in a mifepristone group [27]. Another trial with 25 subjects published in Japan showed that one CM formula, Toki-shakuyaku-san, improved symptoms of hypermenorrhea, dysmenorrhea, and anemia [28]. The same group of researchers has also reported that CM elevates hemoglobin levels in patients with iron-deficiency anemia [29]. Other studies that show the effectiveness of CM on uterine fibroids are either of low quality or contain methodological flaws, such as a lack of randomization, not being blind, or using improper data management [30]. The present study provided epidemiological evidence showing different outcomes between CM users and nonusers among fibroid patients. This study elucidated the possible effect of CM on uterine fibroids from the point of view of public health, expressing the same idea with the above-mentioned clinical studies. 

As the database used by this study was produced primarily for administrative and insurance claim purposes and not for research, the first limitation of this study included the possibility of errors in register information. Secondly, it is also possible that there might have been slight differences in the principle of treatment between hospitals. This might account for the difference in the risk of surgery and CM usage among different areas. Thirdly, the NHI does not cover infertility, meaning that most infertility patients were not registered in the NHI database, leading to possible bias in our data related to infertility. Lastly, the most important disadvantage of studies which extract data from the NHI database is that the causal relationship cannot be clarified. The negative correlation between surgery and the use of CM could come from the effect of CM or the original rejection of surgery by CM users. Therefore, further rigorous, randomized, multicentered, and double-blinded placebo-controlled clinical trial should be carried out to study the efficacy of CM.

## 5. Conclusion

The present study revealed that the risk and the incidence of uterine surgery were lower in CM users than CM nonusers among patients with uterine fibroids, implying an effective treatment of fibroid-related symptoms provided by CM. The low incidence of surgery in CM users existed in almost all investigated sociodemographic and comorbid subgroups. Further large clinical trials are obviously required to evaluate the dimension of benefits that CM could provide. If the protective effect described herein is confirmed, CM is worthy of adoption by women with uterine fibroids not only in Asian but also in Western countries.

## Figures and Tables

**Table 1:** Comparison of socio-demographic factors and co-morbidities between Chinese medicine (CM) users and nonusers in patients with uterine fibroids.

	CM Nonusers *N* = 4452		CM Users *N* = 12238		Total *N* = 16690		*P* value^a^
	*n*	%	*n*	%	*n*	%	
Age, years							<0.0001
<20	18	0.40	110	0.90	128	0.77	
20–29	268	6.02	1151	9.41	1419	8.50	
30–39	1120	25.2	3603	29.4	4723	28.3	
40–49	2394	53.8	5643	46.1	8037	48.2	
≥50	652	14.7	1731	14.1	2383	14.3	
Mean ± SD	42.9 ± 8.20		41.6 ± 8.84		41.9 ± 8.69		<0.0001
Income, US$ per month							0.86
<564.3	1178	26.5	3276	26.8	4454	26.7	
564.3–656.5	868	19.5	2438	19.9	3306	19.8	
656.6–779.6	1293	29.0	3514	28.7	4807	28.8	
≥779.7	1113	25.0	3010	24.6	4123	24.7	
Occupational status							0.009
White collar	2454	55.1	7015	57.3	9469	56.8	
Blue collar	1500	33.7	4016	32.8	5516	33.1	
Others	498	11.2	1202	9.83	1700	10.2	
Area							<0.0001
Northern Taiwan	2288	51.4	5505	45.0	7793	46.7	
Central Taiwan	604	13.6	2353	19.2	2957	17.7	
Southern Taiwan	1364	30.6	3852	31.5	5216	31.3	
Eastern Taiwan and offshore islands	196	4.40	523	4.28	719	4.31	
Comorbidity							
Excessive menstruation	782	17.6	2718	22.2	3500	21.0	<0.0001
Iron-deficiency anemia	473	10.6	1549	12.7	2022	12.1	0.0004
Dysmenorrhea	555	12.5	2571	21.0	3126	18.7	<0.0001
Infertility	9	0.20	94	0.77	103	0.62	<0.0001

^a^Chi-square test and *t*-test.

**Table 2:** Crude/adjusted hazard ratios and 95% confidence intervals for undergoing surgery.

	Crude		Adjusted^a^	
	HR	(95% CI)	HR	(95% CI)
CM				
No	1.00	(Reference)	1.00	(Reference)
Yes	0.20	(0.18–0.21)***	0.18	(0.17–0.19)***
Age, years				
<30	1.00	(Reference)	1.00	(Reference)
30–39	2.65	(2.24–3.14)***	2.36	(1.99–2.80)***
40–49	3.00	(2.54–3.54)***	2.54	(2.15–3.00)***
≥50	1.35	(1.11–1.64)**	1.24	(1.02–1.51)*
Occupational status				
White collar	1.00	(Reference)	1.00	(Reference)
Blue collar	1.18	(1.11–1.27)***	1.15	(1.07–1.23)***
Others	0.99	(0.89–1.11)	0.90	(0.81–1.01)
Area				
Northern Taiwan	1.00	(Reference)	1.00	(Reference)
Central Taiwan	1.25	(1.15–1.37)***	1.51	(1.38–1.65)***
Southern Taiwan	1.20	(1.12–1.30)***	1.26	(1.17–1.36)***
Eastern Taiwan and offshore islands	1.09	(0.93–1.29)	1.03	(0.87–1.22)
Co-morbidity (versus no)				
Excessive menstruation	1.34	(1.25–1.44)***	1.26	(1.17–1.36)***
Iron-deficiency anemia	1.61	(1.48–1.75)***	1.56	(1.43–1.70)***
Dysmenorrhea	1.23	(1.14–1.32)***	1.35	(1.25–1.47)***
Infertility	0.68	(0.12–1.09)	—	

^a^Adjusted for age, occupation, area, excessive menstruation, iron-deficiency anemia, and dysmenorrhea.

**P < 0.01 and ***P < 0.0001.

**Table 3:** Incidence and relative incidence of surgery for CM users and nonusers.

	CM nonusers			CM users			IRR^b^	(95% CI)
	Patients with surgery	Person-years	IR^a^	Patients with surgery	Person-years	IR
Overall	2074	19994	103.73	1607	90744	17.71	0.17	(0.16–0.18)***
Age, years								
<20	0	144	0.00	0	843	0.00	—	
20–29	66	1578	41.83	84	8849	9.49	0.23	(0.16–0.31)***
30–39	509	5390	94.43	636	26484	24.01	0.25	(0.23–0.29)***
40–49	1281	9488	135.01	807	41316	19.53	0.14	(0.13–0.16)***
≥50	218	3394	64.23	80	13252	6.04	0.09	(0.07–0.12)***
Income, US$ per month								
<564.3	516	5401	95.54	406	24169	16.80	0.18	(0.15–0.20)***
564.3–656.5	415	3854	107.68	334	18075	18.48	0.17	(0.15–0.20)***
656.6–779.6	671	5268	127.37	469	26095	17.97	0.14	(0.13–0.16)***
≥779.7	472	5470	86.29	398	22406	17.76	0.21	(0.18–0.24)***
Occupational status								
White collar	1094	11524	94.93	899	52095	17.26	0.18	(0.17–0.20)***
Blue collar	779	6078	128.17	559	29825	18.74	0.15	(0.13–0.16)***
Others	201	2392	84.03	149	8824	16.89	0.20	(0.16–0.25)***
Area								
Northern Taiwan	946	11161	84.76	618	41349	14.95	0.18	(0.16–0.20)***
Central Taiwan	361	2137	168.93	368	17166	21.44	0.13	(0.11–0.15)***
Southern Taiwan	682	5759	118.42	549	28345	19.37	0.16	(0.15–0.18)***
Eastern Taiwan and offshore islands	85	935	90.91	72	3884	18.54	0.20	(0.15–0.28)***
Comorbidity								
Excessive menstruation								
No	1653	16616	99.48	1056	71005	14.87	0.15	(0.14–0.16)***
Yes	421	3378	124.63	551	19739	27.91	0.22	(0.20–0.25)***
Iron-deficiency anemia								
No	1800	18054	99.70	1225	79864	15.34	0.15	(0.14–0.17)***
Yes	274	1940	141.24	382	10880	35.11	0.25	(0.21–0.29)***
Dysmenorrhea								
No	1776	17481	101.60	1072	72183	14.85	0.15	(0.14–0.16)***
Yes	298	2512	118.63	535	18562	28.82	0.24	(0.21–0.28)***
Infertility								
No	2072	19929	103.97	1592	90011	17.69	0.17	(0.16–0.18)***
Yes	2	65	30.77	15	733	20.46	0.67	(0.15–2.91)

^a^IR: incidence rate per 1,000 people.

^b^IRR: incidence rate ratio, compared to CM nonusers.

***P < 0.0001.

**Table 4:** Incidence for women who received myomectomy among time lag.

Diagnosis-to-surgery period (year)	CM nonusers			CM users			cHR^a^	(95% CI)	aHR^b^	(95% CI)
	Patients with surgery	Person-years	IR	Patients with surgery	Person-years	IR
Overall	2074	19994	103.73	1607	90744	17.71	0.20	(0.18–0.21)***	0.18	(0.17–0.19)***
>0.25	882	19922	44.27	1483	90732	16.34	0.38	(0.35–0.41)***	0.33	(0.30–0.36)***
>0.5	731	19863	36.80	1416	90705	15.61	0.43	(0.39–0.47)***	0.37	(0.34–0.41)***
>1	562	19730	28.48	1285	90601	14.18	0.50	(0.45–0.55)***	0.42	(0.38–0.47)***
>3	253	19060	13.27	871	89670	9.71	0.72	(0.63–0.83)***	0.59	(0.51–0.68)***
≥5	91	18346	4.96	455	87840	5.18	1.01	(0.80–1.26)	0.79	(0.63–0.99)*

^a^cHR: crude HR.

^b^aHR: HR adjusted for age, occupation, area, excessive menstruation, iron-deficiency anemia, and dysmenorrhea.

*P < 0.05 and ***P < 0.0001.

## Abbreviations

CI: Confidence interval
CM: Chinese medicine
ICD-9-CM: International Classification of Diseases, 9th Revision, Clinical Modification
HR: Hazard ratio
IR: Incidence rate
IRR: Incidence rate ratio
LHID: Longitudinal Health Insurance Database
NHI: National Health Insurance.
